# Association of disrespectful care after childbirth and COVID-19 exposure with postpartum depression symptoms- a longitudinal cohort study in Nepal

**DOI:** 10.1186/s12884-023-05457-0

**Published:** 2023-03-04

**Authors:** Ashish KC, Ankit Acharya, Pratiksha Bhattarai, Omkar Basnet, Anisha Shrestha, Garima Rijal, Alkistis Skalkidou

**Affiliations:** 1 School of Public Health and Community Medicine, Sahlgrenska Academy, University of Gothenburg, Gothenberg, Sweden; 2 Department of Women’s and Children’s Health, Uppsala University, SE-751 85 Uppsala, Sweden; 3 Research Division, Golden Community, Lalitpur, Nepal

**Keywords:** Immediate breast feeding, Skin to skin contact, Disrespectful care after birth, Postpartum depression, Nepal

## Abstract

**Background:**

The COVID-19 pandemic has led to unprecedented mental stress to women after childbirth. In this study, we assessed the association of disrespectful care after childbirth and COVID-19 exposure before/during labour with postpartum depression symptoms assessed at 7 and 45 days in Nepal.

**Methods:**

A longitudinal cohort study was conducted in 9 hospitals of Nepal among 898 women. The independent data collection system was established in each hospital to collection information on disrespectful care after birth via observation, exposure to COVID-19 infection before/during labour and other socio-demographic via interview. The information on depressive symptoms at 7 and 45 days was collected using the validated Edinburg Postnatal Depression Scale (EPDS) tool. Multi-level regression was performed to assess the association of disrespectful care after birth and COVID-19 exposure with postpartum depression.

**Result:**

In the study, 16.5% were exposed to COVID-19 before/during labour and 41.8% of them received disrespectful care after childbirth. At 7 and 45 days postpartum, 21.3% and 22.4% of women reported depressive symptoms respectively. In the multi-level analysis, at the 7th postpartum day, women who had disrespectful care and no COVID-19 exposure still had 1.78 higher odds of having depressive symptom (aOR, 1.78; 95% CI; 1.16, 2.72). In the multi-level analysis, at 45^th^ postpartum day, women who had disrespectful care and no COVID-19 exposure had 1.37 higher odds of having depressive symptoms (aOR, 1.37; 95% CI; 0.82, 2.30), but not statistically significant.

**Conclusion:**

Disrespectful care after childbirth was strongly associated with postpartum depression symptoms irrespective of COVID-19 exposure during pregnancy. Caregivers, even during the global pandemic, should continue to focus their attention for immediate breast feeding and skin-to-skin contact, as this might reduce the risk for depressive symptoms postpartum.

**Supplementary Information:**

The online version contains supplementary material available at 10.1186/s12884-023-05457-0.

## Background

The number of people affected by depression worldwide is as high as 264 million [1]. The global prevalence of postpartum depression is estimated to be 15%, while in developing countries the 20% of women are affected [2, 3]. Postpartum mental health status is of particular concern as women undergo a surge of hormonal change, family and social adaption [4, 5]. Despite the increase in the institutional birth in low- and middle-income countries since the beginning of millennium development goal period, there has been wide scale reporting of poor experience of care to women during childbirth and postpartum period [6, 7]. Women report being abused and disrespected during childbirth and postpartum period [8, 9]. These poor experience of care during childbirth might increase the vulnerability towards mental health deterioration [10].

Women who are socially disadvantaged report more disrespect during childbirth than women who are relatively advantaged [11, 12]. In Nepal, more than 80% of the women report disrespected after birth i.e. in term of no opportunity to discuss any concerns with health care provider and lack of adequate information. Young women are more likely to be disrespected than older aged women [13]. Women from a relatively disadvantaged (Dalit) ethnic group were more likely to be mistreated compared to a relatively advantaged (Chettri) ethnic group. Most importantly, the health care settings where the number of deliveries is disproportionately high to number of health care providers (HCP), the reporting of disrespectful care has been high [14].

COVID-19 pandemic has caused wide scale unprecedented disruption to routine care to women during childbirth and postpartum period [15]. The pandemic related disruption has led to reduced access to labour and delivery care. Simultaneously diversion of HCP to COVID-19 management has severely constrained the routine health system for a small number of HCP to take care of women [14, 16]. This has exacerbated the disrespectful care during childbirth. Further the fear of risk of COVID-19 infection to women during pregnancy and childbirth might increase the anxiety and postpartum depression [17]. Studies during the pandemic have reported increase in postpartum anxiety and depression associated with lockdown [18–20]. Since, both the disrespectful care during childbirth and postpartum depression is exacerbated during pandemic, there is a need to understand the causal pathway of pandemic disruption to postpartum depression. Further understanding whether the exposure of women to COVID-19 infection before and during labour is associated with postpartum depression is required.

Using the existing longitudinal cohort of women, we assessed disrespectful care during birth and exposure to COVID-19 infection before /and during labour and association of these two exposures to postpartum depression on 7 and 45 days.

## Method

### Study design and setting

A prospective longitudinal cohort design was implemented. The study was conducted in 9 hospitals, where quality improvement projects SUSTAIN [21] and REFINE [22] projects were implemented.

### Study participants

Women who were giving childbirth vaginally and had fetal heart sound during childbirth were eligible for the study. Women were informed and written consent was obtained. Women who had liveborn neonates were included for clinical observation. Women who had stillbirth and neonate with congenital malformation were excluded.

### Sample size

We randomly selected 10% of the women with live born during the first six months of pandemic to estimate adequate number women who might be at risk of postpartum depression. This is based on the estimate that 20% of women have postpartum depression [23]. A total of 2022 women were consented to participate in this study and 898 women-infant pair intrapartum care was observed. These women were followed up at 7 and 45 days postpartum, and depression symptoms were assessed using the Edinburgh Postpartum Depression Scale (EPDS).

#### Data collection

A team of maternal and child health specialists devised and developed the structured questionnaire, and also trained the data collectors before the data collection started (supplementary file 1 and 2). The questionnaire was tested and implemented in the large-scale projects [21, 22]. The data collector informed the eligible women about the study and enrolled those who provided written consent data surveillance team collected the data for the study.

The telephone follow-up was conducted at 7 and 45 days after childbirth. Thus, every participant was contacted 3 times during the study period. Demographic information including their full contact details were collected. They were contacted through telephone phone follow up to collect data on socio-demographic background, obstetric characteristics, disrespectful care after childbirth, sense of coherence and postpartum depression using a tablet-based application.

## Measurements

### Outcome

Depressive symptoms at 7 and 45 days- Using the validated freely available Edinburgh Postnatal Depression Scale (EPDS), the score 10 or more was categorized as depressive symptoms and score 0–9 as non-depressive symptoms [24, 25].

### Exposures

Disrespectful care after birth- defined as the absence of breastfeeding within delivery room and/or absence of skin-to-skin contact with mother immediately after birth (please see exact procedure below, under data analysis).

Exposure to COVID-19 before or/and during labour- defined as women reporting on exposure or coming in contact with people infected with COVID-19 before or/and during labour.

### Other variables

#### Sense of Coherence (SOC)

Olsson et al  [26] was assessed by a 13-item questionnaire, SOC-13 [27], which measures resilience in terms of psychological wellbeing, social wellbeing, social support and factors like stress and adaptive coping strategies during postpartum period. The score of less than 60 was categorized as low SOC and 60 or more was categorized as high SOC.

#### Ethnicity

The ethnicity is the social class system in Nepal mainly categorized herein as women from relatively socially advantaged and relatively socially disadvantaged group. Women from Chettri-Brahmin ethnic group are categorized as relatively socially advantaged and Janjati, muslim, Madeshi and Dalit ethnic group as relatively disadvantaged group.

#### Maternal age

 Age categories for women included 18 years or less, 19–24 years, 25–29 years, 30–34 years, and over 35 years old.

#### Maternal education level

The maternal education was classified into two categories: not educated (illiterate and able to read and write) and educated, including those who had primary, secondary or higher secondary level of education.

#### Parity

Parity was categorized as nulliparous (women who had no previous births), primiparous (women with at least one previous birth) and multiparous (women having two or more previous births).

#### Mode of childbirth

Categorized as spontaneous vaginal and assisted vaginal birth.

### Gestational age

 Based on the last menstrual cycle count; preterm birth was defined as birth occurring prior to 37 weeks of gestation.

#### Birth weight

Birth weight of the newborn was classified as normal birth weight (birth weight ≥ 2500 g) and low birth weight (birth weight < 2500 g). We defined sex of the newborn as either male or female.

### Data management and analysis

Data analysis was conducted using IBM SPSS Statistics Version 26 and STATA 2.1.1. Among the women-infant pairs observed, coverage of immediate breast feeding (IBF) and newborn kept skin to skin (STS) contact after birth was assessed. Based on the coverage of women and newborn who did not receive IBF and STS, we combined and constructed a continuous score to represent disrespectful care after birth. This score was created during the principal component analysis (PCA) of the two indicators. The usual practice is to weight the index according to the first principal component i.e. the component which has the highest variance. The continuous score is more flexible to analyse and to model. We consider the first principal component as the proxy for the disrespectful care index as it explains more than forty-four percent of the total variation. A continuous score of respectful care between -1.78 to + 1.78 was generated. The continuous score was dichotomized as respectful care if score is more than 0 and disrespectful care if less than 0.

Based on the respectful/disrespectful care after birth and COVID-19 exposure, coming from high vs low incidence COVID-19 area, the study participants were categorized into four different groups. These four groups were those who received 1) respectful care after birth with no COVID-19 exposure (reference category), 2) respectful care after birth with COVID-19 exposure, 3) disrespectful care after birth with no COVID-19 exposure and 4) disrespectful care after birth with COVID-19 exposure. Rates of positive screening for postpartum depression at 7 and 45 days in the four groups were assessed. The association between postpartum depression and socio-demographic, obstetrics and neonatal characteristics was assessed using bivariate logistic regression. Further modelling with logistic regression was conducted to assess the association of combinations of disrespectful care during childbirth and COVID-19 exposure with postpartum depression. Model I include unadjusted odds ratio; model II- factors impacting on both exposure and outcome (confounders) and model III- factors impacting on both exposure and outcome, or those impacting only on outcome (some are a result of the exposure, and thus can act as mediators).

In this study, we considered ethnicity, age, maternal education level, birth weight, mode of delivery and parity as confounding variables; disrespectful care at birth and COVID-19 as exposure variables; and postpartum depression as the outcome variable. SOC in this model acts as mediator, since it is affected by the exposure variables and affects the outcome variable i.e., postpartum depression. The data is made available in the supplementary file (supplementary file 3).

## Results

**Fig. 1:**
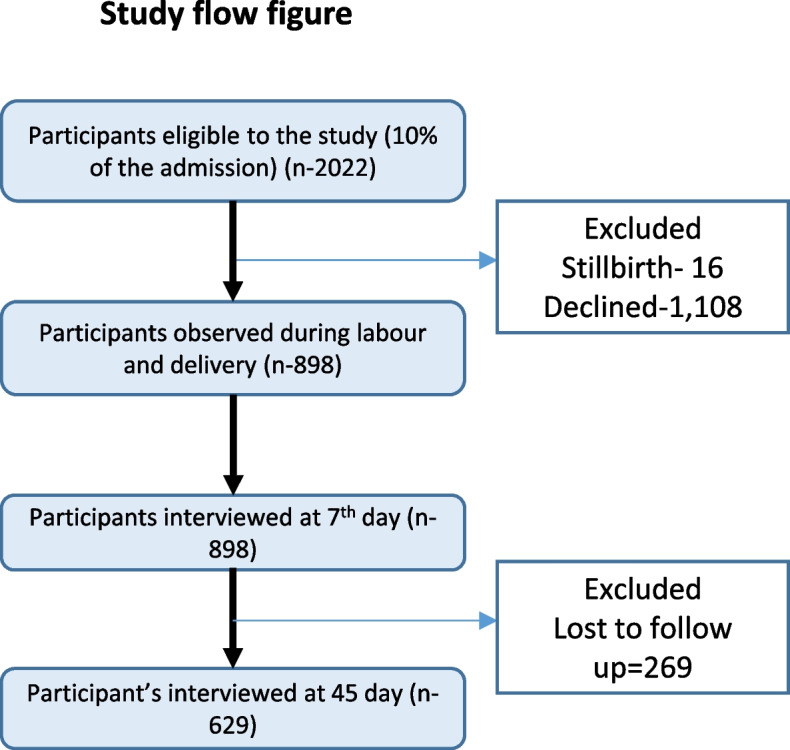
Study flow figure

A total of 2022 women were eligible for the study, of whom 898 were observed during labour and were followed up. On 7^th^ day, 898 women and on 45^th^ day 629 women were reached (Fig. 1).

Among the 898 women-newborn pair observed, 58.2% of them had respectful care i.e. having immediate breast feeding at birth and newborn kept skin to skin contact with mother. Of the women enrolled 16.5% (148/898) of them were exposed to COVID-19 before or/during labour. There was no significant association between women exposed to COVID-19 and disrespectful care after birth (44.6% vs 41.2%, cOR, 1.15; 95% CI; 0.81, 1.64) (Table 1).

**Table 1:** Association of women exposure to COVID-19 before/during labour and disrespectful care after birth

	Respectful care (523, 58.2%)	Disrespectful (375, 41.8%)	cOR (95% CI)
Women's exposure to COVID-19			
No (750)	441 (58.8%)	309 (41.2%)	Reference
Yes (148)	82 (55.4%)	66 (44.6%)	1.15 (0.81, 1.64)

**Fig. 2:**
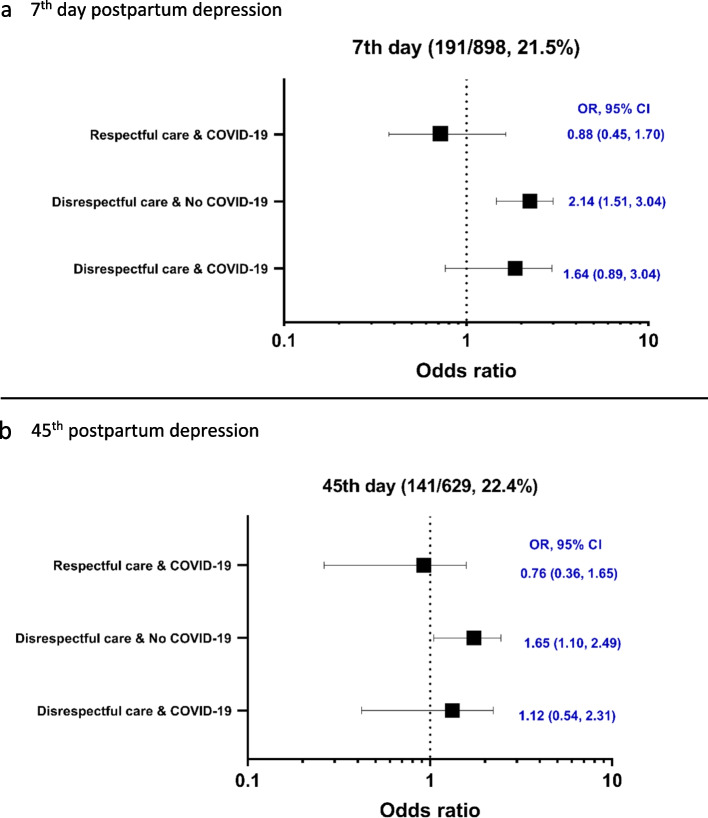
Unadjusted Odds Ratios with 95% CI for associations between disrespectful care after birth and COVID-19 exposure and significant depressive symptoms on 7^th^ and 45^th^ day postpartum*. **a** 7^th^ day postpartum depression2. **b** 45.^th^ postpartum depression *reference- Respectful care and no COVID-19

At the 7th day postpartum, 21.3% of women reported depressive symptoms. In reference to women who had respectful care and no COVID-19 exposure, those who had disrespectful care and no COVID-19 exposure had 2.14 higher odds of having depressive symptoms (cOR, 2.14; 95% CI; 1.51, 3.04); those had disrespectful care and COVID-19 exposure had no significant risk to depressive symptoms (cOR, 1.64; 95% CI; 0.89, 3.04) and those having respectful care after birth and COVID-19 exposure had no significant risk to depressive symptoms (cOR, 0.88; 95% CI; 0.45, 1.70) (Fig. 2a).

At the 45th day postpartum, 22.4% of women reported depressive symptoms. In reference to those who had respectful care at birth and no COVID-19 exposure, women who had disrespectful care and no COVID-19 exposure had 1.65 higher odds of having depressive symptoms (aOR, 1.65; 95% CI; 1.10, 2.49); those had disrespectful care and COVID-19 exposure had no significant risk to depressive symptoms (cOR, 1.12; 95% CI; 0.54, 2.31) and those having respectful care after birth and COVID-19 exposure had no significant risk to depressive symptoms (cOR, 0.76; 95% CI; 0.36, 1.65) (Fig. 2b).

In the multi-level analysis, in model II, after controlling for confounders, at the 7^th^ day postpartum, in reference to women who had respectful care at birth and no COVID-19 exposure, women who had disrespectful care and no COVID-19 exposure still had 1.78 higher odds of having depressive symptom (aOR, 1.78; 95% CI; 1.16, 2.72). The same was true after further adjusting for possible moderators in Model III (aOR, 1.78; 95% CI; 1.15, 2.71). At 45th day, associations were no longer statistically significant in the adjusted analyses (Tables 2 and 3).

**Table 2:** Association between combinations of disrespectful care and COVID-19 exposure and postpartum depression symptoms at 7 days after birth, adjusted also for relevant socio-demographic, obstetric and neonatal characteristics

	No depressive symptom (707)	Depressive symptom (191)	^b^Model I; cOR, 95% CI	^c^Model II; aOR 95% CI	^d^Model III; aOR, 95% CI
Disrespectful care and COVID-19 exposure					
Respectful care and No COVID-19 (441)	369 (83.7%)	72 (16.3%)	Reference	Reference	Reference
Disrespectful care and COVID-19 (66)	50 (75.8%)	16 (24.2%)	1.64 (0.89, 3.04)	1.56 (0.75, 3.27)	1.67 (0.79, 2.53)
Disrespectful care and No COVID-19 (309)	218 (70.6%)	91 (29.4%)	2.14 (1.51, 3.04)	1.78 (1.16, 2.72)	1.78 (1.15, 2.71)
Respectful care and COVID-19 (82)	70 (85.4%)	12 (14.6%)	0.88 (0.45, 1.70)	0.89 (0.40, 1.98)	0.94 (0.42, 2.11)
Maternal education^a^					
Educated (559)	454 (81.2%)	105 (18.8%)	Reference	Reference	Reference
Not educated (98)	56 (57.1%)	42 (42.9%)	3.24 (2.06, 5.10)	2.01 (1.23, 3.29)	2.00 (1.22, 3.29)
Ethnicity					
Advantaged (255)	229 (89.8%)	26 (10.2%)	Reference	Reference	Reference
Disadvantaged (643)	478 (74.3%)	165 (25.7%)	3.04 (1.95, 4.73)	2.41 (1.42, 4.09)	2.38 (1.40, 4.04)
Maternal age					
Less than 18 (40)	30 (75.0%)	10 (25.0%)	1.12 (0.53, 2.37)		
19–24 years (463)	357 (77.1%)	106 (22.9%)	Reference		
25–29 years (283)	235 (83.0%)	48 (17.0%)	0.69 (0.47, 1.01)		
30–34 years (84)	63 (75.0%)	21(25.0%)	1.12 (0.66, 1.93)		
35 year or more (28)	22 (78.6%)	6 (21.4%)	0.92 (0.36, 2.32)		
Parity					
No previous birth (317)	272 (85.8%)	45 (14.2%)	0.67 (0.45, 1.02)	0.74 (0.45, 1.22)	0.78 (0.47, 1.28)
One previous birth (355)	285 (80.3%)	70 (19.7%)	Reference	Reference	Reference
More than one previous birth (226)	150 (66.4%)	76 (33.6%)	2.06 (1.41, 3.02)	1.73 (1.09, 2.74)	1.69 (1.06, 2.68)
Mode of birth					
Assisted birth (35)	31 (88.6%)	4 (11.4%)	Reference		Reference
Spontaneous Vaginal (861)	674 (78.3%)	187 (21.7%)	2.15 (0.75, 6.17)		0.56 (0.18, 1.75)
Preterm					
No (855)	671 (78.5%)	184 (21.5%)	Reference		Reference
Yes (43)	36 (83.7%)	7 (16.3%)	0.71 (0.31, 1.62)		0.71 (0.26, 1.97)
Low Birth Weight					
No (710)	564 (79.4%)	146 (20.6%)	Reference		
Yes (188)	143 (76.1%)	45 (23.9%)	1.22 (0.83, 1.78)		
Sex of baby					
Boy (494)	380 (76.9%)	114 (23.1%)	1.27 (0.92, 1.76)		
Girl (404)	327 (80.9%)	77 (19.1%)	Reference		
Sense Of Coherence					
60–74 (287)	466 (76.3%)	145 (23.7%)	Reference		Reference
< 60 (611)	241 (84.0%)	46 (16.0%)	1.63 (1.13, 2.35)		1.82 (1.11, 2.97)

^a^missing 241

^b^Model I, unadjusted odds ratio

^c^Model II, confounding factors associated with depressive symptom

^d^Model III, confounding and mediating factors associated with depressive symptom

**Table 3:** Association between combinations of disrespectful care and COVID-19 exposure and postpartum depression symptoms at 45 days after birth, adjusted also for relevant socio-demographic, obstetric and neonatal characteristics

	No depressive symptom (488)	Depressive symptom (141)	^b^Model I; cOR, 95% CI	^c^Model II; aOR 95% CI	^d^Model III; aOR, 95% CI
Disrespectful care and COVID-19 exposure					
Disrespectful care and COVID-19 (66)	41 (78.8%)	11 (21.2%)	1.12 (0.54, 2.31)	0.85 (0.34, 2.06)	0.90 (036, 2.28)
Disrespectful care and No COVID-19 (309)	161 (71.6%)	64 (28.4%)	1.65 (1.10, 2.49)	1.41 (0.85, 2.34)	1.37 (0.82, 2.30)
Respectful care and COVID-19 (82)	49 (84.5%)	9 (15.5%)	0.76 (0.36, 1.65)	0.85 (0.33, 2.21)	0.90 (0.34, 2.38)
Respectful care and No COVID-19 (441)	237 (80.6%)	57 (19.4%)	Reference	Reference	Reference
Maternal education^a^					
Educated (428)	350 (81.8%)	78 (18.2%)	Reference	Reference	
Uneducated (83)	35 (42.2%)	48 (57.8%)	6.15 (3.73, 10.1)	3.64 (2.08, 6.34)	3.80 (2.11, 6.86)
Ethnicity					
Advantaged (175)	159 (90.9%)	16 (9.1%)	Reference		Reference
Disadvantaged (454)	329 (72.5%)	125 (27.5%)	3.78 (2.17, 6.57)	2.89 (1.50, 5.56)	2.54 (1.29, 5.01)
Maternal age					
Less than 18 (23)	15 (65.2%)	8 (34.8%)	2.29 (0.93, 5.65)	1.21 (0.39, 3.77)	1.21 (0.36, 4.13)
19–24 years (323)	262 (81.1%)	61 (18.9%)	Reference	Reference	
25–29 years (198)	148 (74.7%)	50 (25.3%)	1.45 (0.95, 2.22)	1.28 (0.78, 2.09)	0.89 (0.51, 1.55)
30–34 years (65)	45 (69.2%)	20 (30.8%)	1.91 (1.05, 3.46)	1.16 (0.57, 2.37)	0.72 (0.33, 1.57)
35 year or more (20)	18 (90.0%)	2 (10.0%)	0.48 (0.11, 2.11)	0.16 (0.02, 1.35)	0.10 (0.01, 0.87)
Parity					
No previous birth (198)	185 (93.4%)	13 (6.6%)	0.26 (0.14, 0.48)	0.25 (0.12, 0.51)	0.26 (0.12, 0.54)
1 previous birth (255)	200 (78.4%)	55 (21.6%)	Reference		
2 or more previous birth (176)	103 (58.5%)	73 (41.5%)	2.58 (1.69, 3.94)	1.97 (1.20, 3.25)	2.19 (1.28, 3.75)
Mode of birth					
Spontaneous vaginal birth (600)	461 (76.8%)	139 (23.2%)	Reference		
Assisted Vaginal (27)	25 (92.6%)	2 (7.4%)	0.27 (0.06, 1.13)		0.23 (0.04, 1.33)
Preterm					
No (597)	457 (76.5%)	140 (23.5%)	Reference		Reference
Yes (32)	31 (96.9%)	1 (3.1%)	0.11 (0.01, 0.78)		0.11 (0.01, 0.90)
Low Birth Weight					
No (491)	370 (75.4%)	121 (24.6%)	Reference		
Yes (138)	118 (85.5%)	20 (14.5%)	0.52 (0.31, 0.87)		
Sex of baby					
Girl (272)	217 (79.8%)	55 (20.2%)	1.25 (0.85, 1.84)		
Boy (357)	271 (75.9%)	86 (24.1%)	Reference		
Sense of Coherence Score					
60–74 score (142)	130 (91.5%)	12 (8.5%)	Reference		
Less than 60 score (487)	358 (73.5%)	129 (26.5%)	3.90 (2.09, 7.29)		3.99 (1.84, 8.68)

^a^118

^b^Model I, unadjusted odds ratio;

^c^Model II, confounding factors associated with depressive symptom;

^d^Model III, confounding and mediating factors associated with depressive symptom

### Other important correlates

 Importantly, at all time-points, factors still being statistically significantly associated with depression in model III were disadvantaged ethnicity, low education, high parity and low sense of coherence (Tables 2 and 3).

## Discussion

This study reports that at more than one in five women may be at risk of having depressive symptoms during the early postpartum period and can persist long after the period. In the first 7 days of birth, disrespectful care after birth (no skin to skin contact and immediate breast feeding) had higher risk of postpartum depression. In the 45^th^ days of birth, disrespectful care consistently remained a risk factor for postpartum depression. Both in 7^th^ and 45^th^ day, the study did not find significant association between COVID-19 exposure before/or during labour with postpartum depression. Other important factors increasing risk for depression at all time-points postpartum were found to be low sense of coherence, disadvantaged ethnic group and no education.

In the hospitals where the study was conducted, the national policy to improve quality of intrapartum and postpartum care was implemented as part of SUSTAIN and REFINE program [21, 22]. Since stepped wise implementation was done in these hospitals, the adequacy of implementation of immediate breast feeding and skin to skin contact differs by hospital. The study was conducted during the middle of COVID-19 pandemic, the human resources were diverted from the labour and delivery room to COVID-19 ward and the coverage for immediate newborn care was inadequate and heterogenous [14].

Our results corroborate with the systematic review, which estimated one in five women in low- and middle-income countries develop postpartum depression [23]. Women from disadvantageous ethnic backgrounds and lacking formal education were at higher risk of developing depressive symptoms [28–30]. Respectful care at birth such as skin-to-skin contact immediately after birth decreases the risk of maternal depression and anxiety and increases the chances for successful breastfeeding [31, 32]. Similarly, higher prevalence of depressive symptoms was reported among those mothers who did not breastfeed their babies than among women who breastfed or only planned to breastfeed [33]. Women exposed to disrespect and abuse during childbirth are more likely to suffer from postpartum depression [10, 34] and these findings are in line with the current study.

Another important exposure to COVID-19 before/or during labour, which might risk for postpartum depression. Women from poor socio-economic backgrounds were possibly more likely to be misinformed about COVID-19, thus may suffer from higher anxiety and depressive symptoms. National lockdown might have perpetuated a sense of danger and uncertainty towards accessing health services, leading to symptoms of maternal depression. Moreover, a restrictive travel policy and restrictions on self-isolation may in the long run lead to a more passive lifestyle and deteriorated mental health [29]. COVID-19 has negatively influenced women's birth satisfaction, as well as increased their symptoms of depression in the postpartum period [35].

Our findings point to the fact that disrespectful care after birth remains the major factors for postpartum depression, especially in the early and late postpartum, even in the middle of a global pandemic. Immediate health interventions must be considered, in order to prevent the deterioration of maternal psychological health during COVID-19 pandemic [36]. Government of Nepal has established mental health counselling services in public hospitals; however, the services were not prioritized for mental health screening and counseling for postpartum women [37]. Moreover, the routine mental health counseling services halted during the pandemic as other routine health services [38]. There is need to establish a community based mental health screening and counseling through the well-established female community health volunteer [39] specially to support women during pandemic lockdown.

### Methodological consideration

Maintaining a cohort of women and infant pair who were observed during childbirth during the COVID-19 pandemic is one of the major strengths of the study. The major limitation is we did not measure the antepartum depression among the women which is a strong correlate with postpartum depression. All women were not tested for COVID-19 infection, so we donot know whether they were infected before and during childbirth. Finally, we had some significant drop out of women during the 45^th^ day of postpartum period.

## Conclusion

This study highlights the relatively strong impact of respectful care, in order to prevent postpartum depression. To minimize the long-term impact of maternal depression, it is essential for hospitals to focus to establish skin-to-skin contact immediately after birth, breastfeeding within one hour and provide proper counseling services. In order to better prepare for future pandemics, sustained quality improvement efforts to ensure adherence to respectful care during birth is important. During the pandemic and emergencies, pregnant and postpartum women are most susceptible to abuse and disrespectful care, thus emergency preparedness to sustain respectful care will be key.

## Supplementary Information

**Additional file 1.**

**Additional file 2.**

**Additional file 3.**

## Data Availability

The dataset generated and analysed is available and provided herein.

## Abbreviations

UNICEF: United Nations Children's Fund
WHO: World Health Organization
SUSTAIN: Scaling Up Safer Birth Bundle Through Quality Improvement in Nepal
REFINE: Rapid Feedback for quality Improvement in Neonatal rEsuscitation
EPDS: Edinburgh Postnatal Depression Scale
SOC: Sense of Coherence
IBF: Immediate Breast Feeding
STS: Immediate Skin to skin contact
cOR: Crudes Odds Ratio
aOR: Adjusted Odds Ratio
