# Supplementary material for: Association of disrespectful care after childbirth and COVID-19 exposure with postpartum depression symptoms- a longitudinal cohort study in Nepal
Source: BMC Pregnancy Childbirth. 2023 Mar 4;23:145. doi: 10.1186/s12884-023-05457-0 (PMC9985076; doi:10.1186/s12884-023-05457-0)
Supplement: Supplementary file 1 — Additional file 1. [file 12884_2023_5457_MOESM1_ESM.docx]

**Form 1**

**SUSTAIN Registry Data**

| **Data Collector details** | | | Name | |  | |
| --- | --- | --- | --- | --- | --- | --- |
|  |  |  | Code | | \|  \|  \|  \|  \|  \|  \|  \|  \| \| --- \| --- \| --- \| --- \| --- \| --- \| --- \| --- \| | |
|  | | | | | | |
| **Data ID** | **Information** | | | **Write or circle where applicable** | | **Remarks** |
| **PART A: BACKGROUND INFORMATION** | | | | | | |
|  | Mother’s first name | | | |  |  |
|  | Mothers’ last name | | | |  |  |
|  | Mother’s inpatient number | | | | \|  \|  \|  \|  \|  \|  \|  \|  \| \| --- \| --- \| --- \| --- \| --- \| --- \| --- \| --- \| |  |
|  | Age of mother (completed years) | | | | \|  \|  \| \| --- \| --- \| |  |
|  | Ethnicity code | | | | Dalit………………………………………………….1  Janajati………………………………………………2  Madhesi……………………………………………..3  Muslim………………………………………………4  Brahmin/Chhetri…………………………………….5  Others……………………………………………….6 |  |
|  | Address | Province | | |  |  |
|  |  | District | | |  |  |
|  |  | Municipality | | |  |  |
|  |  | Ward | | |  |  |
|  | Mobile | | | | \|  \|  \|  \|  \|  \|  \|  \|  \|  \|  \| \| --- \| --- \| --- \| --- \| --- \| --- \| --- \| --- \| --- \| --- \| |  |
| 108a | Place of referall for Delivery | | | | Primary health centre…………………..1  District Hospital…………………………2  Health post………………………………3  Other ……………………………………4  NR………………………………………..99  Self……………………………………….5 |  |
|  | Parity | | | | Primipara (0 previous births)………...………….….1 |  |
|  |  |  |  |  | Multipara (2-5 births)..……………….......................2 |  |
|  |  |  |  |  | Grand multipara (>5 births)………….......................3 |  |

|  | Date (AD) dd/mm/yyyy | Signature |
| --- | --- | --- |
| Form completed: |  |  |
| Data entered into database: |  |  |

| **PART B: PRE-DELIVERY DETAILS** | | | | | | | | | |
| --- | --- | --- | --- | --- | --- | --- | --- | --- | --- |
| **Part B1: Complications during pregnancy (based on ANC records)** | | | | | | | | | |
|  | **Complications recorded** | | | ***Yes*** | | ***No*** | | ***NR*** |  |
| 109a. | *Vaginal bleeding/APH* | | | 1 | | 0 | | 99 |  |
| 109b. | *Decreased foetal movements* | | | 1 | | 0 | | 99 |  |
| 109c. | *Eclampsia (including convulsions, coma, stroke or unconsciousness)* | | | 1 | | 0 | | 99 |  |
| 109d. | *Pre-eclampsia (BP >140/9mmHg and protein urine)* | | | 1 | | 0 | | 99 |  |
| 109e. | *Diabeties* | | | 1 | | 0 | | 99 |  |
| 109f. | *TORCHES* | | | 1 | | 0 | | 99 |  |
| 109g | *TORCHES (specify)………………………………* | | |  | |  | |  |  |
| 109h. | *Others (specify) ………………………………* | | |  | | | | |  |
|  | Place making referral | | |  | | | | |  |
|  | Anaemia | | | Yes……………..…………....1  No………………………...…2  Not recorded……….……..99 | | | | | **If No or NR, go to 114** |
|  | If Yes, type of anaemia | | | Mild (<11 gm/dL)………….…1  Moderate (7-10.9 gm/dL)…..2  Severe (<7 gm/dL)………….3 | | | | |  |
|  | Cause of anaemia (multiple choice) | | | Iron deficiency……………...1  Folic acid deficiency………..2  Vitamin B12 deficiency……..3  Sickle cell…………………...4  Thalassemia………………...5  Others (specify)…………..…. | | | | |  |
| Height | Height of the mother in cm | | | …………………. CM | | | | |  |
| Weight | Weight of the mother from 1^st^ ANC checkup | | | …………………….KG’s | | | | |  |
| **Part B2: Condition at the time of admission** | | | | | | | | | |
|  | **Provisional diagnosis of any complication recorded at the time of admission** | | ***Yes*** | | ***No*** | | ***NR*** | |  |
| 114a. | *Antepartum haemorrhage (APH)* | | 1 | | 0 | | 99 | |  |
| 114b. | *Decreased foetal movements* | |  | |  | |  | |  |
| 114c. | *Prolonged labour (>12 hours active phase)* | | 1 | | 0 | | 99 | |  |
| 114d. | *Pre-eclampsia (BP >140/9mmHg and protein urine)* | | 1 | | 0 | | 99 | |  |
| 114e. | *Eclampsia (including convulsions, coma, stroke or unconsciousness)* | | 1 | | 0 | | 99 | |  |
| 114f. | *Breech or transverse lie* | | 1 | | 0 | | 99 | |  |
| 114g. | *Prolapsed cord* | | 1 | | 0 | | 99 | |  |
| 114h. | *Chorioamnionitis* | | 1 | | 0 | | 99 | |  |
| 114i. | *Premature rupture of membrane (PROM)* | | 1 | | 0 | | 99 | |  |
| 114j. | *Pre-term labour and/or preterm premature rupture of membranes (PPROM)* | | 1 | | 0 | | 99 | |  |
| 114k. | *Foetal congenital anomaly* | | 1 | | 0 | | 99 | |  |
| 114l. | *Others (specify):* | |  | | | | | |  |
|  | Gestational age at admission by USG | | \|  \|  \| + \|  \| \| --- \| --- \| --- \| --- \| \| Not known…………………......…0  Not recorded……………............99 \| \| \| \| \| | | | | | |  |
| 115A | Gestational age at admission by LMP | | \|  \|  \| + \|  \| \| --- \| --- \| --- \| --- \| \| Not known…………………......…0  Not recorded……………............99 \| \| \| \| \| | | | | | |  |
| Q115B | Anemia diagnosed during admission | | Yes……………..…………....1  No………………………...…2  Not recorded……….……..99 | | | | | |  |
| Q115C | If Yes, type of anaemia | | Mild (<11 gm/dL)………….…1  Moderate (7-10.9 gm/dL)…..2  Severe (<7 gm/dL)………….3 | | | | | |  |
| Q115D | Cause of anaemia (multiple choice) | | Iron deficiency……………...1  Folic acid deficiency………..2  Vitamin B12 deficiency……..3  Sickle cell…………………...4  Thalassemia………………...5  Others (specify)…………….6  recorded …………………..99 | | | | | |  |
|  | Mother’s blood group | | RH positive…………………….…..1  RH negative……………………….2  Not recorded…….........................99 | | | | | |  |
|  | ABO blood type | | A……………………………………1  B……………………………………2  AB………………………………….3  O……………………………….…..4 | | | | | |  |
|  | FHR at admission | | Yes, normal (100-160 bpm)………..1  Yes, abnormal (<110 or >160 bpm)..2  Absent…..……………..…..............3  Not recorded…….........................99 | | | | | | **If No or NR, go to 120** |
|  | If FHR recorded | Date (AD) dd/mm/yyyy | \|  \|  \|  \|  \|  \|  \|  \|  \| \| --- \| --- \| --- \| --- \| --- \| --- \| --- \| --- \| | | | | | |  |
|  |  | Time (hh:mm) 24-hr | \|  \|  \|  \|  \| \| --- \| --- \| --- \| --- \| | | | | | |  |
| 119B | Total Number of PV examinations | |  | | | | | |  |
|  | Stage of labour | | Not in labour…………………..…..1  Latent stage of labour………....….2  First stage of active labour….........3  Second stage of labour……...........4  Third stage of labour………...........5 | | | | | |  |
| 119A | Duration of active stage of labor | | …………………. hours | | | | | |  |

| **PART C: DELIVERY DETAILS** | | | |
| --- | --- | --- | --- |
|  | Place of delivery | Home………………………..…......1  On the way ……………………......2  Health facility…………...…….…...3 | **If Home or On the way, go to 129** |
| Q121a | If Health facility | Inside delivery room……………..1  Outside delivery room…………2  NR………………………………..99 |  |
|  | Partograph use | Yes, completely filled……….….…1  Yes, partially filled……………..….2  Not filled…………………….…….0 |  |
|  | FHR monitoring recorded during delivery | Yes, as per protocol..…….….……1  Yes, sporadically (> once)………...2  Yes, only once………..…………...3  Not recorded………………........99 |  |
|  | Abnormal FHR detected during labour | Yes, bradycardia (< 110)…………..1  Yes, tachycardia (> 160)………….2  Yes, repetitive (during > 50% of contractions) or prolonged (> 3 min) decelerations……………….3  No……………………….………...0  Not recorded……………….…...99 |  |
|  | Induction of labour | Induction with prostaglandins…....1  Induction with amniotomy……….2  Induction with oxytocin…………..3  No……………………….………...0  Not recorded……………….…...99 |  |
|  | Augmentation of labour | Augmentation with oxytocin….…1  Augmentation with amniotomy....2  No……………………….………...0  Not recorded……………….…...99 |  |

|  | Received prophylactic antibiotics | Yes…………………….……………...……..…1  No……………………………......................…0 |  |
| --- | --- | --- | --- |
| 127A | Corticosteroids given to mother  (Dexamethasone/Betamethasone) | Yes…………………….……………...……..…1  No……………………………......................…0 |  |
|  | Mode of delivery | Spontaneous vaginal……………...……….…1  ***Go to 131***  Instrumental…………...………..…............…2  ***Go to 130***  Manoeuvre delivery……………………….....3  ***Go to 131***  Emergency CS……………………………......4  Elective CS………….………..............……….5  ***Go to 129*** |  |
|  | Reason for CS (multiple response) | Foetal distress (FD)………………….………..1  Cephalopelvic disproportion (CPD)……….....2  Abnormal lie/malpresentation/ malposition...3  APH or intrapartum haemorrhage ……….….4  PIH (Pre-eclampsia/eclampsia)………….…....5  Non-progress of labour (NPOL) or prolonged labour………………………………..…....…..6  Multiple pregnancy………....................…......7  Cord around the neck…………………….….8  Cord prolapse……………………...……...….9  Oligohydramnios………………...………..…10  Previous CS…...…………………..........….....11  Maternal request……………………..…......12  Other (specify)………………….……..…..…. |  |
|  | Reason for instrumental delivery | Prolonged labour………….…………….…....1  Foetal distress…….……………….…….…...2  Maternal distress……………….……….…...3  Other (specify)…..……………...…………..…. |  |
|  | Multiple delivery | Yes…………………….……………...……..…1  No……………………………......................…0 |  |
| ***If multiple deliveries, use additional form for each baby!*** | | | |
|  | Delivery conducted by | Nursing staff……………………………..……1  Doctor………………………..……………..…2  Other health personnel…...………………….3  Not recorded…………………………….….99 |  |
|  | Complication to mother before or at the time of delivery | Yes……………………………..…….…….….1  No…………………………….…….………...0  Not recorded…………………………..…...99 | **If No or NR, go to 135** |
|  | If Yes (multiple choice) | Manoeuvre delivery……………….…………1  Prolonged labour…………………………….2  Antepartum haemorrhage…………………..3  Postpartum haemorrhage…………………..4  Pregnancy induced hypertension.. ………....5  Oligohydramnios…………………………….6  Others (specify)…………… ………..………. |  |

|  | Amniotic fluid | Clear…………………………...…….1  Thin meconium stained………...…..2  Thick meconium stained…….....…..3  Not recorded…………...…….…...99 | | | | | |  |
| --- | --- | --- | --- | --- | --- | --- | --- | --- |
|  | Complication to baby at the time of delivery | Yes………………………….…….….1  No……………………...….………...0  Not recorded…………...…….…...99 | | | | | | **If No or NR, go to 138** |
|  | If Yes (multiple choice) | Birth asphyxia……………….……...1  Grunting……………..………..…….2  Foetal distress………...…..…..……3  Preterm………...…..…………..…..4  Meconium stained…………...….....5  Tachypnoea………...…..………..…6  Breech delivery……………..….…..7  Low birth weight……………...…...8  Sepsis………………..………….….9  Cyanosis……………..………….…10  Poor cry………………….………...11  Respiratory distress syndrome…...12  Big baby…………………………...13  Gasping …………………..…..…...14  Others (specify)……………..………. | | | | | |  |
|  | Date of delivery (AD) dd/mm/yyyy | \|  \|  \|  \|  \|  \|  \|  \|  \| \| --- \| --- \| --- \| --- \| --- \| --- \| --- \| --- \| | | | | | |  |
|  | Time of delivery (hh:mm) 24-hr | \|  \|  \|  \|  \| \| --- \| --- \| --- \| --- \| | | | | | |  |
|  | Sex of baby | Girl…………………………......….....1  Boy……………….............................0 | | | | | |  |
|  | Birth weight (grams) | \|  \|  \|  \|  \| \| --- \| --- \| --- \| --- \| | | | | | |  |
|  | Gestational age by LMP (weeks) | \|  \|  \| + \|  \| \| --- \| --- \| --- \| --- \| \| Not known………………….......…0  Not recorded…………….............99 \| \| \| \| \| | | | | | |  |
|  | Delivery outcome | Livebirth………………..……….......1  Stillbirth………...…………………...2 | | | | | | **If Livebirth, go to 145** |
|  | If stillbirth, type of stillbirth | Fresh……………………….…...…...1  Macerated……………………......…2 | | | | | | **End extraction!!!** |
|  | APGAR at 1 minute | \|  \|  \| \| --- \| --- \| | | | | | |  |
|  | APGAR at 5 minutes | \|  \|  \| \| --- \| --- \| | | | | | |  |
|  | **Method of Resuscitation** | ***Yes*** | | ***No*** | | ***NR*** | |  |
| 147a. | Clearing of airway (suctioning) | 1 | | 0 | | 99 | |  |
| 147b. | Stimulation | 1 | | 0 | | 99 | |  |
| 147c. | Bag-mask ventilation | 1 | | 0 | | 99 | |  |
|  | ***Other interventions*** |  | | | | | |  |
| 147d. | Oxygen | 1 | | 0 | | 99 | |  |
| 147e. | Medication | 1 | | 0 | | 99 | |  |
| 147f. | Chest compression | 1 | | 0 | | 99 | |  |
| 147g. | Intubation | 1 | | 0 | | 99 | |  |
| 147h. | Others (specify)……………………… | 1 | | 0 | | 99 | |  |
|  | Malformation | 1 | | 0 | | 99 | | **If No or NR, go to 150** |
|  | If Yes (multiple response) | | Neural tube defects……………….1  Cleft lip……….……………………2  Cleft palate………………………..3  Club foot…………………………..4  Hypospadias………………………5  Omphalocele……………...………6  Gastroschisis………………………7  Imperforate anus…………………8  Other limb defects………………..9  Others (specify)…………………… | | | | |  |
|  | ***Routine care of newborn*** | | ***Yes*** | | ***No*** | | ***NR*** |  |
|  | Vitamin K | | 1 | | 0 | | 99 |  |
|  | Body temperature | | 1 | | 0 | | 99 |  |
|  | Respiratory rate | | 1 | | 0 | | 99 |  |
|  | Medical examination of baby | | 1 | | 0 | | 99 |  |
|  | Newborn transferred from labour room | | Yes, SNCU/NICU………………......1  Yes, PNC…………………………..2  No……….……………………..….0  Not recorded…….………….......99 | | | | | **If SNCU/NICU, go to Part D** |
|  | Outcome of the baby | | Healthy…………………………….1  Improved……………………….....2  Referred to other facility…….…...3  **Died…..……………………………**4  Absconded………………...……...5  Stillbirth…………………………...7  DOPR……………………………...8 | | | | | **If other than died goto q158** |
|  | Date of death | | \|  \|  \|  \|  \|  \|  \|  \|  \| \| --- \| --- \| --- \| --- \| --- \| --- \| --- \| --- \| | | | | |  |
|  | Cause of death (multiple response) | | Neonatal sepsis/infection…………1  Meconium aspiration syndrome….2  Birth asphyxia……………………..3  Respiratory distress syndrome…..4  Hypoglycaemia……………………5  Low birth weight (LBW)………….6  Preterm……………………………7  Congenital malformation………...8  Others (specify)…………………….. | | | | |  |
|  | Date of discharge (AD) dd/mm/yyyy | | \|  \|  \|  \|  \|  \|  \|  \|  \| \| --- \| --- \| --- \| --- \| --- \| --- \| --- \| --- \| | | | | |  |

***Note: Fill this section for sick newborns only!***

| **PART D: SICK NEWBORN INFORMATION** | | | | | | | | | | | | | | |  |  |
| --- | --- | --- | --- | --- | --- | --- | --- | --- | --- | --- | --- | --- | --- | --- | --- | --- |
| Sicknewborn_ age | | Age of the Sick new born | | | …………………………….. Days | | | | | | | | |  |  |  |
|  | | Sick newborn’s inpatient number | | | \|  \|  \|  \|  \|  \|  \|  \|  \| \| --- \| --- \| --- \| --- \| --- \| --- \| --- \| --- \| | | | | | | | | |  |  |  |
|  | | Date of admission (AD) dd/mm/yyyy | | | \|  \|  \|  \|  \|  \|  \|  \|  \| \| --- \| --- \| --- \| --- \| --- \| --- \| --- \| --- \| | | | | | | | | |  |  |  |
| DeliveryDate | | Date of Delivery (AD) dd/mm/yyyy | | | \|  \|  \|  \|  \|  \|  \|  \|  \| \| --- \| --- \| --- \| --- \| --- \| --- \| --- \| --- \| | | | | | | | | |  |  |  |
| 160B | | Sex of baby | | | Girl………………………….1  Boy…………………………0 | | | | | | | | |  |  |  |
|  | | Weight of the baby at admission | | | \|  \|  \|  \|  \| Grams \| \| --- \| --- \| --- \| --- \| --- \| | | | | | | | | |  |  |  |
|  | | Neonate delivered at this hospital | | | Yes……………………………..…..……1  No……………………………........……0 | | | | | | | | |  |  |  |
|  | | Birth order number | | | Single or Twin 1……………….………..1  Twin 2………………………….…….…2 | | | | | | | | |  |  |  |
| ***If multiple deliveries, use separate forms for each baby!*** | | | | | | | | | | | | | | |  |  |
|  | | Corticosteroids given because of threatening premature birth | | Yes…………………………………...….1  No………………………….…………...0  Not recorded………………………....99 | | | | | | | | | | **If No or NR, go to 166** |  |  |
|  | | If Yes, how many doses? | | \|  \|  \| \| --- \| --- \| | | | | | | | | | |  |  |  |
|  | | **Clinical features** | | **Yes** | | | | **No** | | | | **NR** | |  |  |  |
|  | | Not feeding well | | 1 | | | | 0 | | | | 99 | |  |  |  |
|  | | Seizures | | 1 | | | | 0 | | | | 99 | |  |  |  |
|  | | Lethargic | | 1 | | | | 0 | | | | 99 | |  |  |  |
|  | | Unconscious | | 1 | | | | 0 | | | | 99 | |  |  |  |
|  | | Fast breathing (>60 breaths per min) | | 1 | | | | 0 | | | | 99 | |  |  |  |
|  | | Grunting | | 1 | | | | 0 | | | | 99 | |  |  |  |
|  | | Severe chest indrawing | | 1 | | | | 0 | | | | 99 | |  |  |  |
|  | | Apnoea | | 1 | | | | 0 | | | | 99 | |  |  |  |
|  | | Raised temperature (>38°C) | | 1 | | | | 0 | | | | 99 | |  |  |  |
|  | | Hypothermia (<36.5°C) | | 1 | | | | 0 | | | | 99 | |  |  |  |
|  | | Central cyanosis | | 1 | | | | 0 | | | | 99 | |  |  |  |
|  | | Jaundice | | 1 | | | | 0 | | | | 99 | |  |  |  |
|  | | Abdominal distension | | 1 | | | | 0 | | | | 99 | |  |  |  |
|  | | Congenital anomalies | | Yes…………………………………...….1  No………………………….…………...0  Not recorded………………………....99 | | | | | | | | | | **If No or NR, go to Part E** |  |  |
|  | | If Yes, what congenital anomalies? (multiple choice) | | Neural tube defects………………....….1  Cleft lip………………………...….….....2  Cleft palate………………………....…..3  Club foot………………………...……...4  Hypospadias………………...………….5  Omphalocele……………...………...….6  Gastroschisis………………………...….7  Imperforate anus…………………...….8  Others (specify)………………………… | | | | | | | | | |  |  |  |
| **PART E: TREATMENT AT SNCU/NICU** | | | | | | | | | | | | | | |  |  |
|  | Alternative feeding if baby unable to breastfeed | | | | | Yes…………………………….....…1  No……………………………….....0  Not recorded…………………….99 | | | | | | | **If No or NR, go to 183** | |  |  |
|  | If Yes, mention the type of feeding | | | | | Cup feeding………………………..1  Bottle feeding……………………..2  Spoon feeding…………………….3  NG tube feeding…………………..4 | | | | | | |  | |  |  |
|  | If preterm or LBW, additional feeding | | | | | Yes…………………………….....…1  No……………………………….....0Not recorded…………………….99 | | | | | | | **If No or NR, go to 185** | |  |  |
|  | If Yes, specify | | | | |  | | | | | | |  | |  |  |
|  | Kangaroo Mother Care (KMC) initiated | | | | | Yes…………………………….....…1  No……………………………….....0  Not recorded…………………….99 | | | | | | |  | |  |  |
|  | | | | | | ***Yes*** | | | ***No*** | | ***NR*** | |  | |  |  |
|  | Safe administration of oxygen | | | | | 1 | | | 0 | | 99 | |  | |  |  |
|  | Pulse oximeter used for titration of oxygen | | | | | 1 | | | 0 | | 99 | |  | |  |  |
|  | **Injectable antibiotics for neonatal sepsis as per protocol** | | | | | ***Yes*** | | | ***No*** | | ***NR*** | |  | |  |  |
| 188a. | Ampicillin | | | | | 1 | | | 0 | | 99 | |  | |  |  |
| 188b. | Gentamicin | | | | | 1 | | | 0 | | 99 | |  | |  |  |
| 188c. | Amikacin | | | | | 1 | | | 0 | | 99 | |  | |  |  |
| 188d. | Cefotaxime | | | | | 1 | | | 0 | | 99 | |  | |  |  |
| 188e. | Flucloxacillin | | | | | 1 | | | 0 | | 99 | |  | |  |  |
| 188f. | Ceftazidime | | | | | 1 | | | 0 | | 99 | |  | |  |  |
| 188g. | Vancomycin | | | | | 1 | | | 0 | | 99 | |  | |  |  |
| 188h. | Meropenem | | | | | 1 | | | 0 | | 99 | |  | |  |  |
| 189A | C-Reactive Protien | | | | | Yes, positive…….………………….1  Yes, negative………...…………….2  No………………………………….0  Not recorded…………………….99 | | | | | | |  | |  |  |
|  | Blood culture available | | | | | Yes, positive…….………………….1  Yes, negative………...…………….2  No………………………………….0  Not recorded…………………….99 | | | | | | | **If other than positive, go to 191** | |  |  |
|  | If positive, specify the organism | | | | | Streptococcus……………………..1  Staphylococcus……………………2  Klebsiella…………………………..3  Acinetobacter……………………..4  Others (specify)……………………. | | | | | | |  | |  |  |
|  | | | | | | ***Yes*** | ***No*** | | | ***NR*** | | |  | |  |  |
|  | Management of shock | | | | | 1 | 0 | | | 99 | | |  | |  |  |
|  | Intravenous fluid | | | | | 1 | 0 | | | 99 | | |  | |  |  |
|  | Management of hypoglycaemia | | | | | 1 | 0 | | | 99 | | |  | |  |  |
|  | Effective phototherapy | | | | | 1 | 0 | | | 99 | | |  | |  |  |
|  | Continuous positive airway pressure | | | | | 1 | 0 | | | 99 | | |  | |  |  |
|  | Blood transfusion | | | | | 1 | 0 | | | 99 | | |  | |  |  |
|  | Mechanical/assisted ventilation | | | | | 1 | 0 | | | 99 | | |  | |  |  |
|  | Exchange transfusion | | | | | 1 | 0 | | | 99 | | |  | |  |  |
|  | Seizure management (multiple response) | | | | | Yes, Phenobarbital……….... …..…1  Yes, Calcium gluconate……….......2  Yes, Phenytoin…………………….3  Yes, 10% Dextrose…………………4  No……………………………….…0 | | | | | | |  | |  |  |
|  | Warmth management  (multiple response) | | | | | Incubator………….…………..…....1  KMC…….……………………….….2  Radiant warmer……………………3  Regular bed.………………..……...4  No……………………………….…0 | | | | | | |  | |  |  |
|  | Any other drugs for management | | | | | Vitamin K…………………………...1  Tobramycin eye drop……………...2  Ciprofloxacin eye ointment……….3  No……………………………….…0 | | | | | | |  | |  |  |
|  | Any other procedures for diagnosis | | | | | Echocardiogram…………………...1  Chest x-ray…………………………2  Complete blood count…………….3  Random blood sugar……………...4  No……………………………….…0 | | | | | | |  | |  |  |
|  | Final diagnosis of the sick newborn (multiple response) | | | | | Birth asphyxia……………..…….....1  Hypoxic Ischemic Encephalopathy (HIE)/NE……………………………2  Hyper-bilirubinaemia/jaundice…....3  Meconium aspiration syndrome….4  Neonatal sepsis/infection................5  Respiratory distress syndrome/fast breathing…………………………..6  Hypoglycaemia………………….…7  Pneumonia……………..………….8  Meningitis……………..…………..9  UTI/diarrhoea………………..…...10  Umbilical infection…………….….11  Skin infection……………..............12  Seizure/convulsion…...…………..13  IUGR/Low birth weight………......14  Preterm…………………………...15  Eye infection………………….…..16  Others (specify)……………..……. | | | | | | | **If HIE/NE, go to 4203a**  **If Jaundice, go to 203b**  **If other than HIE/NE or Jaundice, go to Part F** | |  |  |
| 4203A | Baby diagnosed with HIE | | | | | Yes……………………………..1  No……………………………..0 | | | | | | | **If no skip to 4203B** | |  |  |
|  | If HIE/NE, check for grading | | | | | Grade I………………………....…...1  Grade II……………………………..2  Grade III……………………...….....3  Not recorded……………………..99 | | | | | | |  | |  |  |
| 4203B | Baby diagnosed with Hyper-billirubinaemia/jaundice | | | | | Yes……………………………..1  No……………………………..0 | | | | | | | **If no skip to 4206** | |  |  |
|  | If Jaundice | | | | | Physiological………..……………...1  Pathological……………..………....2  Not recorded……………………..99 | | | | | | |  | |  |  |
| **PART F: DISCHARGE DETAILS** | | | | | | | | | | | | | | | | |
|  | Outcome of the baby at discharge | | Improved…………………..........................1  Referred to other facilities…..………...….2  Died…………………………………..…....3  Absconded……………………………..….4 | | | | | | | | | | | **If improved, go to 209** | |  |
|  | Date of death (AD) dd/mm/yyyy | | \|  \|  \|  \|  \|  \|  \|  \|  \| \| --- \| --- \| --- \| --- \| --- \| --- \| --- \| --- \| | | | | | | | | | | |  | |  |
|  | Cause of death (multiple response) | | Neonatal sepsis/infection…………………1  Meconium aspiration syndrome………….2  Birth asphyxia……………………………..3  Hypoxic ischaemic encephalopathy (HIE)…………………………………….…4  Respiratory distress syndrome…………...5  Hypoglycaemia……………………………6  Pneumonia…………………………….......7  Meningitis…………………………………8  Seizure/convulsion………………………..9  Low birth weight (LBW)…………………10  Preterm…………………………………...11  Congenital malfunction………………….12 | | | | | | | | | | |  | |  |
|  | Weight of baby at discharge | | \|  \|  \|  \|  \| Grams \| \| --- \| --- \| --- \| --- \| --- \| | | | | | | | | | | |  | |  |
|  | Date of discharge (AD) dd/mm/yyyy | | \|  \|  \|  \|  \|  \|  \|  \|  \| \| --- \| --- \| --- \| --- \| --- \| --- \| --- \| --- \| | | | | | | | | | | |  | |  |
|  | Duration of stay in the hospital | | \|  \|  \|  \| Days \| \| --- \| --- \| --- \| --- \| | | | | | | | | | | |  | |  |
| Delivery_Date | Date of delivery | | DD/MM/YY | | | | | | | | | | |  | |  |
