# Supplementary material for: Association of disrespectful care after childbirth and COVID-19 exposure with postpartum depression symptoms- a longitudinal cohort study in Nepal
Source: BMC Pregnancy Childbirth. 2023 Mar 4;23:145. doi: 10.1186/s12884-023-05457-0 (PMC9985076; doi:10.1186/s12884-023-05457-0)
Supplement: Supplementary file 2 — Additional file 2. [file 12884_2023_5457_MOESM2_ESM.docx]

**lnlvt d~h'/L -lSnlgsn cjnf]sg**_

gd:t] d]/f] gfd======================================================= xf] / xfdL uf]N8]g sDo'lg6Laf6 ;j]{If0f ub}{5f}+ . xfdL o; c:ktfndf cfdf / gjhft lzz' ;DalGw ;j]{If0f ub}{5f}+, h;df cfdf / gjhft lzz' ;DalGw ljljw ;]jfx?sf] u'0f:t/ s:tf] 5 elg k|ToIf cjnf]sg ug]{5f}+ . xfdL o; ;j]{If0fdf tkfO+{sf] ;xeflutfsf] w]/} sb/ u5f}{ . tkfO+{sf] k|ToIf cjnf]sgaf6 ;+sng ePsf] hfgsf/Lx?n] o; c:ktfn tyf ;/sf/nfO{ :jf:Yo ;DaGwL of]hgf th'{df ug{ d2t k'Ug]5 . o; ;j]{If0fsf] nflu ;fwf/0ftof tkfO+{ egf{ eP b]lv aRrf hGdbf;Ddsf] ;Dk"0f{ ljj/0f cjnf]sg ul/g]5 . o; qmddf tkfO{+nfO{ kg{ hfg] c;'ljwf k|lt Ifdfk|fyL{ 5f} . tkfO{+af6 k|fKt ;Dk"0f{ ljj/0fx? clt uf]Ko /flvg] 5g\ / s;}nfO{ klg b]vfO{g] jf lbOg] 5|}g .

of] ;j]{If0fdf ;xefuL x'g] jf gx'g] tkfO{+sf] :j]R5fsf] s'/f xf] . olb tkfO{+nfO{ s'g} klg ;do c;xh dx;'; ePdf jf cjnf]sg u/]sf] lrQ ga'em]df xfdLnfO{ eGg' xf]nf . tkfO{+n] h'g ;'s} ;dodf cjnf]sg 6'+Uofpg cfu|x ug{ ;Sg'x'g]5 . tyflk, cfzf 5 tkfO+{ ;j]{If0fdf ;xeflu x'g' x'g]5 lsgsL tkfO+{n] lng'ePsf] ;]jfsf] u'0f:t/af/] dxTjk"0f{ ;'rgfx? ;+sng ug{sf ;fy} c:ktfnsf] ;]jf ;'wf/sf lglDt ;xof]uL x'g]5 .

**s] d ca cjnf]sg ug{ ;'? u/f}+ <**

cjnf]sg ul/g] dlxnfsf] ;xL =================================== ldlt=============================

cjnf]sgtf{sf] ;xL =================================== ldlt=============================

cjnf]sg ul/g] dlxnfn] lbg cg'dlt lbPsf] ============================================! ⎯→ cjnf]sg ;'? ug]{

cjnf]sg ul/g] dlxnfn] ;xhstf{sf] ;xfotfn] cg'dlt lbPsf]]] ================= @ ⎯→ cjnf]sg ;'? ug]{

dlxnfn] cjnf]sg ug{ lbg gdfg]sf] ==================================================== # ⎯→ cjnf]sg 6'+Uofpg]

cjnf]sg ug]{ cj:yf g/x]sf] ============================================================= $ ⎯→ cjnf]sg 6'+Uofpg]

**Form 6**

**SUSTAIN Clinical Observation Checklist**

| **Data Collector details** | | Name |  | | |
| --- | --- | --- | --- | --- | --- |
|  |  | Code | \|  \|  \|  \|  \|  \|  \|  \|  \| \| --- \| --- \| --- \| --- \| --- \| --- \| --- \| --- \| | | |
|  | Mother’s first name | |  | | |
|  | Mother’s last name | |  | | |
|  | Inpatient number | | \|  \|  \|  \|  \|  \|  \|  \|  \| \| --- \| --- \| --- \| --- \| --- \| --- \| --- \| --- \| | | |
|  | Informed consent taken?  ***(If No, Stop Observation)*** | | Yes……………………………………….…...1  No………………………………………….…0 | | |
|  | | | | |  |
| **Data ID** | **Information** | | | **Write or code where applicable** | **Remarks** |
| **PART A: ACTIVE FIRST STAGE LABOUR ROOM** | | | | | |
| 605 | PV examinations performed before delivery | | | Yes, at least every hour….………….…….…………….1  Yes, >4hours between examinations…………………….....2  Only Once…………………………..3  No…………......………………….…0 |  |
| 605a | Progress of labour monitored per vaginal(PV) examination during active stage of labour at 4 centimeter dilatation | | | Yes………………………………..1  No………………………………..0 |  |
| 605b | If yes, Number of Progress of labour monitored per vaginal(PV) examination during active stage of labour after 4 centimeter dilatation | | | ……………………………….. |  |
| 605c | Progress of labour monitored per vaginal(PV) examination during active stage of labour at 8 centimeter dilatation | | | Yes………………………………..1  No………………………………...0 |  |
| 605d | If yes, Number of Progress of labour monitored per vaginal(PV) examination during active stage of labour after 8 centimeter dilatation | | | …………………………………. |  |
|  | Colour of amniotic fluid at the time of rupture of membrane | | | Clear fluid……………………………1  Thin meconium stained…………….2  Thick meconium stained…………....3  No Color/Nil………………………..4  No rupture of membrane …………5 |  |
|  | Progress of labour recorded in the partograph immediately after monitoring the labour progress | | | Yes…………………………….…..…1  No…………......………………….…0 |  |
|  | Augmentation of labour | | | Yes, with Oxytocin………………..…1  Yes, with Amniotomy………...…..…2  Others (specify)……………………..  No…………......………………….…0 |  |
|  | FHR monitoring conducted during active stage of labour using | | | Moyo doppler….…………...…….…1  Fetoscope.………………….....….…2  Stethoscope……………...…….…...3  CTG…...………………………….…..4  Others (specify)……………………..  No…………......………………….…0 | **If No, go to Part B** |
| Episode 1 | FHS Monitored using Moyo | | | Yes………………………………….1  No…………………………………..0 | **If no go to 611** |
| 609a. | If Moyo doppler used, serial number of Moyo ***(look at the back of the Moyo)*** | | | \|  \|  \|  \|  \|  \|  \|  \| \| --- \| --- \| --- \| --- \| --- \| --- \| --- \| |  |
| 609b | Date of placement of Moyo (DD/MM/YY) | | | ………/………/…………… |  |
| 609c | Time of placement of Moyo (HH:MM) | | | …… : ……. |  |
| 609d | Fetal Heart Sound at 1 minute after placement of moyo (Specify) | | | ……………………………. |  |
| 610 | FHR monitoring technique using Moyo | | | Continuous………………….…...….1  Intermittent……………………...…2  Only once……………………………3 | **If Continuous, go to 612** |
| 610a1 | If Moyo doppler used, serial number of Moyo ***(look at the back of the Moyo)*** | | | \|  \|  \|  \|  \|  \|  \|  \| \| --- \| --- \| --- \| --- \| --- \| --- \| --- \| |  |
| 610a2 | Date of placement of Moyo (DD/MM/YY) | | | ………/………/…………… |  |
| 610a3 | Time of placement of Moyo (HH:MM) | | | …… : ……. |  |
| 610a4 | Fetal Heart Sound after placement of moyo (Specify) | | | ………………………………….. |  |
| Episode3 | FHS Monitored using Moyo | | | Yes…………………………………1  No………………………………….0 | **If no go to 611** |
| 610b1 | If Moyo doppler used, serial number of Moyo ***(look at the back of the Moyo)*** | | | \|  \|  \|  \|  \|  \|  \|  \| \| --- \| --- \| --- \| --- \| --- \| --- \| --- \| |  |
| 610b2 | Date of placement of Moyo (DD/MM/YY) | | | ………/………/…………… |  |
| 610b3 | Time of placement of Moyo (HH:MM) | | | …… : ……. |  |
| 610b4 | Fetal Heart Sound after placement of moyo (Specify) | | | ………………………………….. |  |
| Episode4 | FHS Monitored using Moyo | | | Yes…………………………………1  No………………………………….0 | **If no go to 611** |
| 610c1 | If Moyo doppler used, serial number of Moyo ***(look at the back of the Moyo)*** | | | \|  \|  \|  \|  \|  \|  \|  \| \| --- \| --- \| --- \| --- \| --- \| --- \| --- \| |  |
| 610c2 | Date of placement of Moyo (DD/MM/YY) | | | ………/………/…………… |  |
| 610c3 | Time of placement of Moyo (HH:MM) | | | …… : ……. |  |
| 610c4 | Fetal Heart Sound after placement of moyo (Specify) | | | ………………………………….. |  |
| Episode5 | FHS Monitored using Moyo | | | Yes…………………………………1  No………………………………….0 | **If no go to 611** |
| 610d1 | If Moyo doppler used, serial number of Moyo ***(look at the back of the Moyo)*** | | | \|  \|  \|  \|  \|  \|  \|  \| \| --- \| --- \| --- \| --- \| --- \| --- \| --- \| |  |
| 610d2 | Date of placement of Moyo (DD/MM/YY) | | | ………/………/…………… |  |
| 610d3 | Time of placement of Moyo (HH:MM) | | | …… : ……. |  |
| 610d4 | Fetal Heart Sound after placement of moyo (Specify) | | | ………………………………….. |  |
| Episode6 | FHS Monitored using Moyo | | | Yes…………………………………1  No………………………………….0 | **If no go to 611** |
| 610e1 | If Moyo doppler used, serial number of Moyo ***(look at the back of the Moyo)*** | | | \|  \|  \|  \|  \|  \|  \|  \| \| --- \| --- \| --- \| --- \| --- \| --- \| --- \| |  |
| 610e2 | Date of placement of Moyo (DD/MM/YY) | | | ………/………/…………… |  |
| 610e3 | Time of placement of Moyo (HH:MM) | | | …… : ……. |  |
| 610e4 | Fetal Heart Sound after placement of moyo (Specify) | | | ………………………………….. |  |
| Episode7 | FHS Monitored using Moyo | | | Yes…………………………………1  No………………………………….0 | **If no go to 611** |
| 610f1 | If Moyo doppler used, serial number of Moyo ***(look at the back of the Moyo)*** | | | \|  \|  \|  \|  \|  \|  \|  \| \| --- \| --- \| --- \| --- \| --- \| --- \| --- \| |  |
| 610f2 | Date of placement of Moyo (DD/MM/YY) | | | ………/………/…………… |  |
| 610f3 | Time of placement of Moyo (HH:MM) | | | …… : ……. |  |
| 610f4 | Fetal Heart Sound after placement of moyo (Specify) | | | ………………………………….. |  |
| Episode 8 | FHS Monitored using Moyo | | | Yes…………………………………1  No………………………………….0 | **If no go to 611** |
| 610g1 | If Moyo doppler used, serial number of Moyo ***(look at the back of the Moyo)*** | | | \|  \|  \|  \|  \|  \|  \|  \| \| --- \| --- \| --- \| --- \| --- \| --- \| --- \| |  |
| 610g2 | Date of placement of Moyo (DD/MM/YY) | | | ………/………/…………… |  |
| 610g3 | Time of placement of Moyo (HH:MM) | | | …… : ……. |  |
| 610g4 | Fetal Heart Sound after placement of moyo (Specify) | | | ………………………………….. |  |
| Episode 9 | FHS Monitored using Moyo | | | Yes…………………………………1  No………………………………….0 | **If no go to 611** |
| 610h1 | If Moyo doppler used, serial number of Moyo ***(look at the back of the Moyo)*** | | | \|  \|  \|  \|  \|  \|  \|  \| \| --- \| --- \| --- \| --- \| --- \| --- \| --- \| |  |
| 610h2 | Date of placement of Moyo (DD/MM/YY) | | | ………/………/…………… |  |
| 610h3 | Time of placement of Moyo (HH:MM) | | | …… : ……. |  |
| 610h4 | Fetal Heart Sound after placement of moyo (Specify) | | | ………………………………….. |  |
| Episode 10 | FHS Monitored using Moyo | | | Yes…………………………………1  No………………………………….0 | **If no go to 611** |
| 610i1 | If Moyo doppler used, serial number of Moyo ***(look at the back of the Moyo)*** | | | \|  \|  \|  \|  \|  \|  \|  \| \| --- \| --- \| --- \| --- \| --- \| --- \| --- \| |  |
| 610i2 | Date of placement of Moyo (DD/MM/YY) | | | ………/………/…………… |  |
| 610i3 | Time of placement of Moyo (HH:MM) | | | …… : ……. |  |
| 610i4 | Fetal Heart Sound after placement of moyo (Specify) | | | ………………………………….. |  |
| Episode 11 | FHS Monitored using Moyo | | | Yes…………………………………1  No………………………………….0 | **If no go to 611** |
| 610j1 | If Moyo doppler used, serial number of Moyo ***(look at the back of the Moyo)*** | | | \|  \|  \|  \|  \|  \|  \|  \| \| --- \| --- \| --- \| --- \| --- \| --- \| --- \| |  |
| 610j2 | Date of placement of Moyo (DD/MM/YY) | | | ………/………/…………… |  |
| 610j3 | Time of placement of Moyo (HH:MM) | | | …… : ……. |  |
| 610j4 | Fetal Heart Sound after placement of moyo (Specify) | | | ………………………………….. |  |
| 611 | Approximated average interval between each FHR monitoring conducted during second stage of active labour | | | 5-15 minutes…………………………1  15-30 minutes……………...……..….2  31-45 minutes……..…..………….….3  46-60 minutes………………………4  >60 minutes………..…………….…5 |  |
|  |  | | |  |  |

|  | Date (AD) dd/mm/yyyy | Name & Signature |
| --- | --- | --- |
| Form completed: |  |  |
| Data entered into database: |  |  |

|  | Abnormal FHR detected during labour | Yes, bradycardia (<100)……………...1  Yes, tachycardia (>160)………….…..2  No……………………….…………...0  Absent……………………………….3 | **If No, go to 616** |
| --- | --- | --- | --- |
| 612a | If Bradycardia or Tachycardia | Repetitive (during > 50% of contractions)…………………………1  Prolonged (>3 mins)…………………2 |  |
|  | Time of first abnormal FHR (hh:mm) | \|  \|  \|  \|  \| 24-hr \| \| --- \| --- \| --- \| --- \| --- \| |  |
|  | Time of last abnormal FHR before birth (hh:mm) | \|  \|  \|  \|  \| 24-hr \| \| --- \| --- \| --- \| --- \| --- \| |  |
|  | FHR recorded in partograph immediately after monitoring | Yes…………………………….…..…1  No…………......………………….…0 |  |
|  | Companion at the time of labour | Husband……………..………………1  Mother……………..………………..2  Mother-in-law………………..……...3  Other family members……………...4  Friends/neighbours……...…………..5  None………………………...……….0 |  |

| **PART B: DELIVERY OBSERVATION** | | | |
| --- | --- | --- | --- |
|  | Temperature of delivery room | \|  \| **°C** \| \| --- \| --- \| \| Room thermometer NA…………….0 \| \| |  |
|  | Health worker(s) wash their hands with disinfectants before delivery | Yes……………………………...…..…1  No…………......………………..….…0 |  |
|  | Health worker(s) put on gloves on both hands and gown to prepare for birth | Yes……………………………...…..…1  No…………......………………..….…0 |  |
|  | Prepares necessary supplies and equipment for immediate newborn care  ***Look for*** *– highly disinfected or sterile scissors, cord tie and clean blanket to dry the baby* | Yes……………………………...…..…1  No…………......………………..….…0 |  |
|  | Prepares for resuscitation before delivery  ***Look for*** – *self-inflating bag, masks size 0 and 1, suction device, NeoBeat, stethoscope, gloves* | Yes……………………………...…..…1  No…………......………………..….…0 |  |
|  | Health workers greets the mother  Look for – *whether health worker introduces herself to the mother, informs about the mother’s status at the time of delivery* | Yes……………………………...…..…1  No…………......………………..….…0 |  |
|  | Mode of delivery | Spontaneous vaginal……………..…..1  Instrumental…..………..….….…...…2  Manoeuvre delivery…………...…..…3  Emergency CS……………..….………4  Elective CS………………………….....5 |  |
|  | If CS or instrumental delivery due to foetal distress, time between decision of CS or instrumental delivery until delivery | \|  \|  \| mins \| \| --- \| --- \| --- \| |  |
|  | Shifted to Operation Theatre (OT) due to non-progress of labour | Yes……………………………...…..…1  No…………......………………..….…0 |  |
| ***If Emergency or Elective CS, Conclude Observation!!!*** | | | |
|  | Multiple delivery | Yes…………………………….…....…1  No…………......…………………...….0 |  |
| ***If multiple deliveries, use separate forms for each delivery*** | | | |
|  | Partograph | Completed as per protocol….........….1  Partially completed………..................2  Filled in retrospectively………..…..…3  Not used………………...……….…...0 |  |
|  | Companion at the time of delivery | Husband………………………………1  Mother………………………………..2  Mother-in-law………………………...3  Other family members……………….4  Friends/neighbours…………………..5  None………………………………….0 |  |

| **PART C: ESSENTIAL NEWBORN CARE** | | | |
| --- | --- | --- | --- |
|  | NeoBeat placed immediately after delivery | Yes………………..…………….…...…1  No………………………...…………...0 | **If No, go to 633** |
|  | If Yes, time of placement of NeoBeat | \|  \|  \|  \|  \| 24-hr \| \| --- \| --- \| --- \| --- \| --- \| | **If NeoBeat isn’t used, go to 633** |
|  | ***Heart Rate reading from NeoBeat*** |  |  |
| 632a. | HR at 30 secs | \|  \|  \|  \| bpm \| \| --- \| --- \| --- \| --- \| |  |
| 632b. | HR at 1 min | \|  \|  \|  \| bpm \| \| --- \| --- \| --- \| --- \| |  |
| 632c. | HR at 2 mins | \|  \|  \|  \| bpm \| \| --- \| --- \| --- \| --- \| |  |
| 632d. | HR at 5 mins | \|  \|  \|  \| bpm \| \| --- \| --- \| --- \| --- \| |  |
| 632e. | HR at 10 mins | \|  \|  \|  \| bpm \| \| --- \| --- \| --- \| --- \| |  |
|  | Immediate thorough drying | Yes……………………………...…..…1  No……………………………..……....0 |  |
|  | Crying of the baby immediately after birth | Yes……………………………...…..…1  No……………………………..……....0 | **If No, go to 636** |
|  | Time of cry of the baby (hh:mm) 24-hr | \|  \|  \|  \|  \| \| --- \| --- \| --- \| --- \| | **Go to 646** |
|  | Breathing spontaneously after birth (without stimulation) | Yes……………………………...…..…1  No……………………………..……....0 | **If No, go to 639** |
|  | Time of spontaneous breathing (hh:mm) 24-hr | \|  \|  \|  \|  \| \| --- \| --- \| --- \| --- \| | **Go to 646** |
|  | Secretion present | Yes……………………………...…..…1  No……………………………..……....0 |  |
|  | Health worker cleared airway | Yes……………………………...…..…1  No……………………………..……....0 | If no goto q643 if baby not breathing |
|  | Airway cleared with | Electric suction………...……….....…..1  Penguin suction…...…………...…..…2  Bulb suction…………..........................3  Hand or cloth..……………....….….…4 |  |

|  | | | | ***Yes*** | ***No*** |  |
| --- | --- | --- | --- | --- | --- | --- |
|  | Newborn baby cried immediately after clearing airway | | | 1 | 0 | **If Yes, go to 646** |
|  | Newborn baby breathed immediately after clearing airway | | | 1 | 0 | **If Yes, go to 646** |
|  | Health worker stimulated the baby | | | 1 | 0 |  |
|  | Newborn baby cried immediately after stimulation | | | 1 | 0 | **If Yes, go to 646** |
|  | Newborn baby breathed immediately after stimulation | | | 1 | 0 |  |
|  | Timing of cord clamping | Early (<60 secs)……………………….1  Delayed (60-180 secs)...........................2  Prolonged (>180 secs)……………......3 | | | |  |
|  | Time of cord clamping (hh:mm) 24-hr | \|  \|  \|  \|  \| \| --- \| --- \| --- \| --- \| | | | | **If cord clamped after baby cried, go to 663** |
| Baby_Resus | Baby taken to resuscitation table for bag and mask ventilation | 1 | 0 | | | **Applicable for all** |
|  | Selection of correct mask for ventilation | | | 1 | 0 |  |
|  | Ventilated with bag-mask within 1 minute (60 seconds) | | | 1 | 0 |  |
|  | Ventilation with bag and mask done at the rate of 40-60 breaths per minute | | | 1 | 0 |  |
|  | Rising of chest after each ventilation | | | 1 | 0 | **If Yes, go to 653** |
|  | Improved ventilation | | | 1 | 0 |  |
|  | Newborn baby cried immediately after ventilation | | | 1 | 0 | **If Yes, go to 660** |
|  | Newborn baby breathed immediately after ventilation | | | 1 | 0 | **If Yes, go to 660** |
|  | Called for help | | | 1 | 0 |  |
|  | Someone came for help | | | 1 | 0 |  |
|  | Assessment of newborn heart beat conducted after 1 minute of ventilation | | | 1 | 0 |  |
|  | Additional resuscitation actions performed | | | 1 | 0 | **If No, go to 660** |
|  | If Yes, what resuscitation actions? | Medication………………………….....1  Chest compression…….......................2  Intubation…………………………….3  Oxygen……………………….……….4 | | | |  |
|  | Time when resuscitation stopped (hh:mm) 24-hr | \|  \|  \|  \|  \| \| --- \| --- \| --- \| --- \| | | | |  |
| Q661_Breath | Baby spontaneously breathing? | Yes…………………….1  No…………………….0 | | | | If No goto bagmaskvent |
|  | Time of spontaneous breathing after ventilation (hh:mm) 24-hr | \|  \|  \|  \|  \| \| --- \| --- \| --- \| --- \| | | | |  |
| Bagmask  vent | Type of Bag and Mask used for ventilation | Horizontal………………………..1  Vertical(upright)…………………2 | | | |  |
|  | Outcome of newborn | Livebirth………………………………1  Stillbirth……………………………….2 | | | |  |
| 662a | If Still birth Specify | Fresh………………………………….1  Macerated……………………………2 | | | | **If stillbirth, go to Part D** |
|  | Baby kept in skin-to-skin contact in the mother’s chest in delivery room | Yes……………………………...…..…1  No……………………………..……....0 | | | | **If No, go to 665** |
|  | Duration of skin-to-skin contact | \|  \|  \| mins \| \| --- \| --- \| --- \| | | | |  |
|  | Breastfeeding initiated 1 hour after birth | Yes……………………………...…..…1  No……………………………..……....0 | | | | **If No, go to 667** |
|  | Time of initiation of first breastfeeding (hh:mm) 24-hr | \|  \|  \|  \|  \| \| --- \| --- \| --- \| --- \| | | | |  |
|  | Newborn’s body and head covered during the stay in delivery room | Yes……………………………...…..…1  No……………………………..……....0 | | | |  |

|  | Was the weighing scale calibrated accurately before taking the weight? | Yes……………………………...…..…1  No……………………………..……....0 |  |
| --- | --- | --- | --- |
|  | Birth weight taken by scale within 90 minutes after delivery | Yes……………………………...…..…1  No……………………………..……....0 |  |
|  | Malformation | Yes……………………………...…..…1  No……………………………..……....0 | **If No, go to 672** |
|  | If Yes, type of malformation (multiple choice) | Neural tube defects…………………..1  Cleft lip....……….…………………….2  Cleft palate……………………………3  Club foot………………………………4  Hypospadias…………………………..5  Omphalocele………………………….6  Gastroschisis………………………….7  Imperforate anus………….………….8  Anencephaly…………………………9  Hydrocephalus…………………….10  Diaphragmatic hernia………………...11  Others (specify)………………………12 |  |
|  | Newborn baby received injectable antibiotics | Yes…………………………………..…1  No……………………………..……....0 |  |
|  | Anything applied to the cord | Chlorhexidine........................................1  Nothing applied…………………...….2  Others (specify)…………………….…. | **If Nothing applied, go to 675** |
|  | Time of application (hh:mm) 24-hr | \|  \|  \|  \|  \| \| --- \| --- \| --- \| --- \| |  |
|  | Newborn Vitamin-K injection provided | Yes……………………………...…..…1  No……………………………..……....0 |  |

| **PART D: THIRD STAGE OF LABOR AND POSTNATAL CARE** | | | | | |
| --- | --- | --- | --- | --- | --- |
|  | | | ***Yes*** | ***No*** |  |
|  | Mother received Oxytocin IM injection | | 1 | 0 |  |
|  | Mother received post-delivery antibiotics | | 1 | 0 |  |
|  | Health worker massaged the uterus | | 1 | 0 |  |
|  | Placenta examined after delivery | | 1 | 0 |  |
|  | Placenta weighed | | 1 | 0 |  |
|  | Health worker inspected the lower vagina and perineum for lacerations/tears and repairs lacerations/tears, if necessary | | 1 | 0 |  |
|  | Health worker made sure mother is clean and comfortable | | 1 | 0 |  |
|  | Any of the relatives accompany the mother in the delivery room | | 1 | 0 |  |
|  | Health workers washed their hands with disinfectants after delivery | | 1 | 0 |  |
|  | Health workers wore gloves while taking care of newborn | | 1 | 0 |  |
|  | Administration of Vitamin A | | 1 | 0 |  |
|  | Newborn transferred to | PNC………………...………..1  SNCU/NICU………………....2  Observation room….............3  Referred to higher centre….4  Stillbirth…………………….5  Died…………………………6 | | |  |
